# Effects of *Hibiscus sabdariffa* supplementation on metabolism and bladder in obese rats

**DOI:** 10.1590/acb395024

**Published:** 2024-09-06

**Authors:** Pedro Pajolli, Paulo Roberto Kawano, Hamilto Yamamoto, Rodrigo Guerra, Bruno Matos Moraes, Leonardo Oliveira Reis, Flavio Vasconcelos Ordones, Oscar Eduardo Hidetoshi Fugita, Alessandra Melchert, João Luiz Amaro

**Affiliations:** 1 Universidade Estadual Paulista “Júlio de Mesquita Filho” – Faculdade de Medicina – Divisão de Urologia – Botucatu (SP) – Brazil.; 2 Pontifícia Universidade Católica de Campinas – Departamento de Imunoncologia – Campinas (SP) – Brazil.; 3 Universidade Estadual de Campinas – Department of Surgery – Campinas (SP) – Brazil.; 4 University of Auckland – Department of Surgery – Auckland – New Zealand.; 5 Universidade Estadual Paulista “Júlio de Mesquita Filho” – Faculdade de Medicina Veterinária e Zootecnia – Departamento de Veterinária Clínica – Botucatu (SP) – Brazil.

**Keywords:** Obesity, Metabolism, Interleukins, Hibiscus, Bladder

## Abstract

**Purpose:**

*Hibiscus sabdariffa* (HS) extract has several health benefits and anti-obesogenic effects. The aim of the present study was to assess whether the medicinal properties attributable to HS would prevent or mitigate bladder changes induced by obesity in an experimental model.

**Methods:**

Forty-eight male Wistar rats were submitted to one of four different dietary interventions (12 animals each): G1, standard diet and water (controls); G2, standard diet and HS tea; G3, a palatable high-fat diet and water; and G4, high-fat diet diet and HS tea. The animals were monitored for body weight, feed, and water and tea intake, according to the allocated group. After 16 weeks, the animals were euthanized, and the levels of creatinine, inflammatory cytokines, testosterone, cholesterol, triglycerides, and electrolytes were evaluated. In addition, histopathological analysis of the animals’ bladder was performed.

**Results:**

Groups receiving HS (G2 and G4) showed decreased levels of the pro-inflammatory cytokine interleukin-1α. HS tea was able to reduce low-density lipoprotein and triglyceride levels in the G2 group compared to other groups. Only in the G3 there was a significant increase in the body weight when it was compared the 12^th^ and 16^th^ weeks. Leptin was shown to be elevated in the groups that received a high-fat diet. There was a significant decrease in the muscle fibers thickness and in the total collagen count in G4 bladder when compared with G1 and G3.

**Conclusions:**

HS has an anti-inflammatory role, can reverse hyperlipidemia in rats, and reduced deleterious effects of obesity on these animals’ bladder.

## Introduction

Obesity has become a worldwide issue due to its high incidence and prevalence rates in the overall population. In addition to excess of adipose tissue itself, obesity causes disorders such as increased inflammatory levels, type-2 diabetes, cardiovascular disease, and kidney disorders^1^.

Obesity and metabolic syndrome have a high prevalence in the population and are risk factors for lower urinary tract symptoms, overactive bladder and urinary incontinence, which may lead to deterioration in quality of life^2^^–^ 5. However, simple complementary items added to patients’ daily diet could help improve such symptoms^5^.

Different therapeutic approach would be utilized for obesity treatment, and the first line is behavioral counseling as dietary education and physical exercises in routine habits setting^6^. Natural extract derivatives from plants could be also an attractive option in the obesity approach. Thus, natural bioactive compounds such as phenols and flavonoids, found in some tea and coffee, may be useful in the anti-obesity treatment^7^^,^ 8. *Hibiscus sabdariffa* (HS) is a plant from Malvaceae specimens, originary from India, Malaysia, and Africa. The ingestion of HS extract has several health benefits and anti-obesogenic effects^9^^,^ 10.

HS is rich in natural bioactive compounds, such as phenols and flavonoids, which have been reported as effective in treating obesity^7^^,^ 8 ^,^ 11. Anthocyanins are polyphenolic compounds with multiple properties, such as antioxidant activity and protection against oxidative stress, anti-inflammatory and anti-carcinogenic action, and can help control diabetes and obesity, and prevent cardiovascular diseases^12^^–^ 14. Thus, there are several benefits of HS tea, whether promoting weight loss or protecting against comorbidities stemming from obesity^10^.

Therefore, the aim of the present study was to assess whether the different medicinal properties attributable to HS tea would prevent or mitigate metabolic abnormalities and bladder structural changes induced by obesity in an experimental model.

## Methods

All animal experiments complied with the ARRIVE guidelines and were carried out in accordance with the U.K. Animals (Scientific Procedures) Act of 1986 and associated guidelines, EU Directive 2010/63/EU for animal experiments, or the National Institutes of Health (NIH) guide for the care and use of laboratory animals (NIH Publications No. 8023, revised 1978). The study was approved by the School of Veterinary Medicine and Animal Science Ethics Committee, Universidade Estadual Paulista “Júlio de Mesquita Filho”, Botucatu, SP, Brazil (Protocol no. 235/2012).

Forty-eight male *Wistar* rats, 52 days old, weighing 180–220 g, were randomly allocated into four groups with 12 animals each:

- Control group (G1): rats were fed with commercial rat chow (Presence, Purina) and water *ad libitum*;
- Hibiscus group (G2): animals received commercial rat chow and HS tea in aqueous solution, in a dose of approximately 60 mg·kg^-1^ day^-1^ of polyphenols;
- Obese group (G3): rats were fed with a high-fat diet (HFD) and water *ad libitum*;
- Obese + HS group (G4): animals received HFD and HS tea in aqueous solution in a dose of approximately 60 mg·kg^-1^ day^-1^ of polyphenols.

Soluble HS from suitable commercial laboratory with 2.68% of polyphenol content was used. The tea was daily prepared, according to the methodology recommended by McKay et al^15^. In short, the preparation involved adding 240 mL of hot water at 60 °C to 1.25 grams of HS tea (60 mg/kg/day). The rats were placed in individual cages and maintained under controlled lighting and temperature.

HFD contained commercial rat chow (Presence) plus peanuts, milk chocolate, and sweet biscuit in a proportion of 3:2:2:1. All components of the hyperlipidic diet were ground and blended and consisted of lipids 24%, protein 20%, mineral residues 5%, carbohydrates 41%, and fiber 5.9%. The total energy value of the HFD was 480.3 kcal/100 g, with 35% of calories as fats^16^. Commercial rat chow (Presence) consisted of lipids 4%, protein 23%, mineral residues 10%, carbohydrates 55%, and fiber 7%. The total energy value was 366 kcal/100 g.

Initially (M0 moment), all animals were weighed using a digital scale (first week) and after four, eight, 12, and 16 weeks until the end of the study. All rats were individually placed for 24 hours in metabolic cages at the 8th and the 16th week of the study. Animals were fasted overnight, and blood was collected from the dorsal tail vein of all rats to measure serum creatinine (Cr) levels. Urine was collected, centrifuged, and stored at -20ºC until analysis. Cr was measured in 24-hour urine samples harvested in the metabolic cage.

After 16 weeks (M1 moment), the animals were submitted to general anesthesia with tiletamine/zolazepan (Zoletil) 30 mg·kg^-1^ intramuscularly, and sodium thiopental (Thiopentax) 60 mg·kg^-1^ intraperitoneally^17^. Euthanasia was performed by exsanguination under anesthesia. Blood collection by cardiac puncture was performed. The blood was collected in vacuum tubes without anticoagulant and centrifuged to obtain serum. Serum biochemical–urea, Cr, triglyceride, total cholesterol, low-density lipoprotein (LDL)-cholesterol, high-density lipoprotein (HDL)-cholesterol–,and urinary tests (protein and Cr) were evaluated. The assays were performed using commercially available kits (Labtest, Lagoa Santa, Brazil) in a Labmax progress automatic analyzer (Labtest, Lagoa Santa, MG, Brazil).

Leptin, adiponectin, and interleukin (IL)-8 concentrations were determined in serum using enzyme-linked immunosorbent assay (ELISA) kits (Millipore, St. Charles, MO, United States of America). The entire methodology was carried out in the laboratories of the Experimental Research Unit (UNIPEX) of the Faculty of Medicine of Botucatu, at Universidade EStadual Paulista “Júlio de Mesquita Filho”. Values were reported in nanograms per deciliter (ng/dL) of serum. Measurements of IL-4 and -1α were carried out using the Cytometric Bead Array method using CBA Rat Soluble Protein – Flex Set kits, according to the manufacturer’s instructions.

After collection of individual urine (24 h) samples at weeks 8 and 16 of the study, creatinine clearance (CCr) was calculated from the urinary Cr, serum Cr, 24-h urine volume, and body weight by using Eq. 1:


$$\underset{\left(mL/min/Kg\right)}{Ccr}\;\frac{\text{Urine}Cr(mg/dL)\times \text{Urine volume}(mL)}{\text{Serum}Cr(mg/dL)\times [1000/\text{body weight}(g)]\times 1440(min)}$$ (1)

After surgical removal, the bladder was fixed in 10% buffered formaldehyde for 24 hours. Subsequently, they were kept in 70% alcohol until inclusion in paraffin. The paraffin blocks with bladder tissue were sectioned, obtaining 5-μm-thick transverse sections of the bladder wall, which were stained using the hematoxylin and eosin (HE) and Picrosirius red (PR) technique to assess the muscle and collagen fibers, respectively.

**Figure 1:**
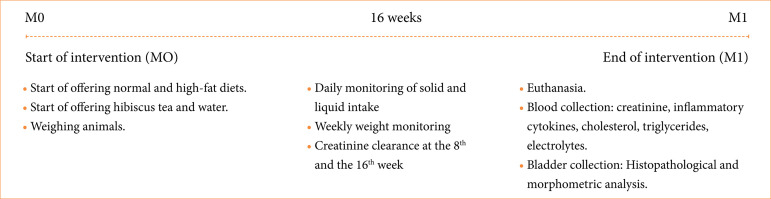
Flowchart demonstrating the different moments and parameters evaluated according to study group.

Source: Elaborated by the authors.

After sectioning the bladder and fixing the slide in HE, muscle fiber measurements were conducted by an experienced pathologist using optical microscopy at 40x magnification. The measurements were performed in the body of the bladder, utilizing the Aperio Imagescope morphometry program. Five random fields were selected, and ten fibers were measured in each field, on a single histological section. Prior to each measurement, the software was calibrated using the scale present in the digitized image provided by the Aperio scanner (Fig. 1).

### Statistical analysis

Data were expressed as means ± standard error. The study of quantitative variables (body weight, serum urea, Cr, and electrolytes, CCr, and lipid profile) considered the model of repeated measurements in a scheme of two factors (diet and HS) and was complemented by Bonferroni multiple-comparison test. Comparison among the body weight measurements in each group was performed using analysis of variance (ANOVA) repeated measures, followed by the Tukey’s post hoc test.

For the histomorphometric analysis, comparisons between groups were made using one-way ANOVA followed by Tukey’s test for variables with normal distribution and Kruskal-Wallis’ test followed by Dunn’s test when variables presented a non-normal distribution (collagen fibers).

The statistical analysis was performed using SigmaStat, version 2.0. Results were considered significant at *p* < 0.05.

## Results

Groups G3 and G4 had a significant lower mean food intake in relation to controls. Initially, we observed an increase in body weight in all animals in different groups, but after the fourth week this gain was significantly higher in groups G3 and G4 compared with the controlled groups (G1 and G2) (Table 1). Only in the G3 there was a significant increase in the body weight when it was compared the 12 and the 16 week (Table 1).

**Table 1:** Animals body weight **(g)** in all groups in different moments (week 1, 4, 8, 12, and 16)*.

Weeks	Groups			
	G1	G2	G3	G4
1	200.5 ± 13.4 ^Aa^	211.5 ± 18.7 ^Aa^	215.9 ± 22.0 ^Aa^	212.2 ± 22.0 ^Aa^
4	359.0 ± 35.0 ^Ba^	371.2 ± 27.1 ^Ba^	412.7 ± 61.8 ^Bb^	420.5 ± 48.7 ^Bb^
8	422.6 ± 43.3 ^Ca^	410.6 ± 38.4 ^Ca^	496.4 ± 83.0 ^Cb^	494.9 ± 67.5 ^Cb^
12	464.8 ± 54.1 ^Da^	441.4 ± 41.2 ^Da^	562.2 ± 93.5 ^Db^	538.8 ± 68.7 ^Db^
16	493.2 ± 52.7 ^Da^	470.4 ± 42,6 ^Da^	616.2 ± 98.9 ^Eb^	591.8 ± 78.9 ^Db^

* Values are expressed as mean ± standard deviation. Different capital letters in the columns indicate statistically significant differences (*p* < 0.05).

Different lowercase letters on the same line indicate statistically significant differences (*p* < 0.05). Source: Elaborated by the authors.

LDL was reduced in G4 compared with G3 (Obese) (5.62 ± 1.19 vs. 7.31 ± 2.29 respectively, *p* < 0.05). The triglyceride levels in G2 were significantly lower in comparison with other groups (G1, G3 and G4) (46.31 ± 15,35 vs. 83.23 ± 34.11, 86.77 ± 36.55, 84.08 ± 32.44 respectively, *p* < 0.01).

24-h CCR was significantly lower in G3 when compared with controls (G1 and G2).

Serum calcium and potassium levels showed no statistically significant difference between the different groups, except for sodium, which showed a slight increase in obese rats treated with HB tea (142.15 ± 1.91 vs. 138.23 ± 2.55 mEq/L; G4 × G1, *p* < 0.05).

Leptin was shown to be elevated in the groups that received HFD (G3 and G4) in comparison to controls (G1 and G2) (Table 2). Groups receiving HB (G2 and G4) showed decreased levels of the pro-inflammatory cytokine IL-1α. There was no statistical difference of the anti-inflammatory cytokines among the groups neither in the different evaluation periods (Table 2).

**Table 2:** Median, minimum, and maximum levels of the cytokines pro-inflammatory (pg/mL) performed on the serum of rats, in the different groups

Cytokine	Groups			
	G1	G2	G3	G4
Leptin	3,157.54* (1,731.8; 8,621.4)	2,750.79# (704.4; 5,006.7)	8,552.62 (2,622.5; 33,347.1)	7,537.30 (3,434,2; 16,091,6)
IL-1α	17.90 (1.03; 52.44)	6.74& (0.50; 220.80)	16.98 (4.76; 47.66)	0.91¥ (1.45; 56.57)
IL-8	30.62 (3.43; 107.07)	17.08 (0.73; 55.92)	25.70 (0.46; 96.25)	20.79 (0.38; 108.58)

* *p* < 0.01: G1 × G3, G4;

# *p* < 0.01: G2 × G3, G4 G1 = G2;

& *p* < 0.05: G2 × G1, G3;

¥ *p* < 0.05: G4 × G1, G2, G3.

Source: Elaborated by the authors.

**Figure 2:**
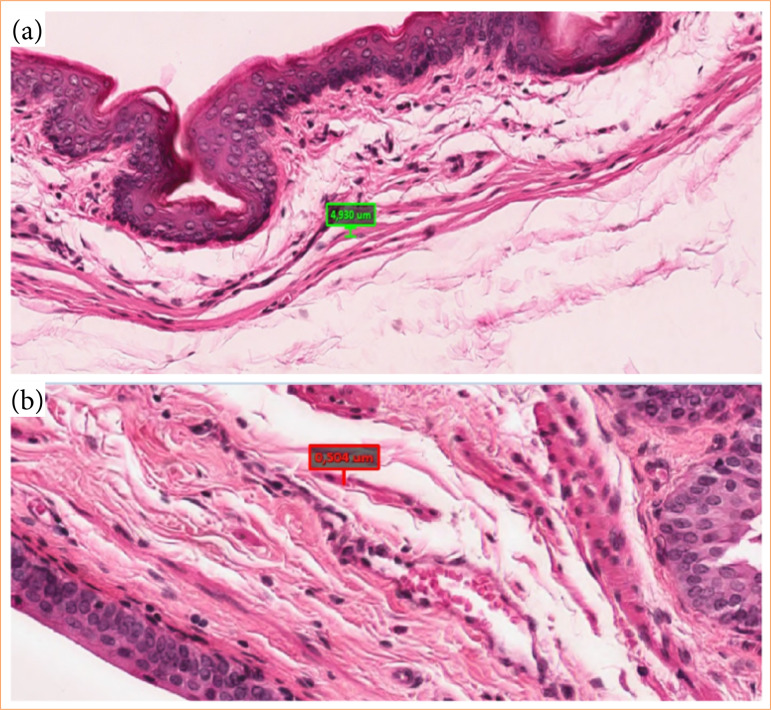
**(a)** Bladder muscular fiber thickness of rats fed with high-fat diet; **(b)** rats with high-fat diet and hibiscus tea. Both images are hematoxylin and eosin stained and 20x magnified.

Source: Elaborated by the authors.

There was a significant decrease in the muscle fibers thickness (Table 3) and in the total collagen count in G4 bladder when compared with G1 and G3 (Table 3, Fig. 2).

**Table 3:** Median, minimum, and maximum values of thickness of the bladder muscle fibers (µm) and total collagen measurement (%), in the different groups*.

	Groups				p-value
	G1	G2	G3	G4	
DMT (µm)	0.84 (0.62–1.15)^ab^	0.64 (0.54–0.80)^bc^	0.50 (0.28–0.79)^ab^	0.36 (0.19–0.54)^c^	0.006
Collagen (%)	15.67 (14.12–23.10)^ab^	11.72 (9.70–15.27)^bc^	17.38 (12.16–22.40)	7.93 (5.79–14.53)^c^	0.01

DMT: Detrusor muscle thickness;

* different lowercase letters on the same line indicate statistically significant differences.

Source: Elaborated by the authors.

## Discussion

Obesity is a multifactorial disease influenced by environmental, genetic, and behavioral factors^18^, which may be associated with an increase in overactive bladder and urinary incontinence^19^^,^ 20. However, a paucity of studies has explored the real mechanisms involved in this bladder dysfunction.

Recent evidence associates the pathogenesis of obesity with a low-intensity systemic inflammatory state, characterized by increased circulating levels of several pro-inflammatory mediators. This could lead to functional and/or morphological bladder changes, possibly accounting for the urinary symptoms observed in these patients^21^. Another possibility would be the association of an increase in body adipose tissue with a reduction in the volume and thickness of the bladder muscle layer^22^, causing an increase in interstitial fibrosis of the detrusor muscle and changes in its contractility^23^.

Therefore, the development of experimental models of obesity can help to understand the behavior of inflammatory and biochemical parameters in obesity. Our model also made it possible to study the anti-inflammatory and lipid-lowering properties of HS tea, and determine whether it has any protective factor against the eventual occurrence of changes in the detrusor muscle of these animals.

There was a significant weight increase in G3 and G4 after the fourth week in comparison with controls, despite a significant lower mean food intake. This fact could be explained by the animals fed with HFD presenting a higher caloric intake and better satiety^16^^,^ 17. Analyzing weight gain in the 16th week, we did not observe a statistically significant difference between G3 and G4, despite a numerical drop in the group supplemented with HS tea, thus demonstrating that HS tea had no relevant action in relation to the protection against obesity in this experiment. This fact is at odds with what has been described by other authors^24^. However, taking into account that in G4 there was no significant weight gain in the 16^th^ week in relation to the previous moment, this may suggest a protective effect of HS tea in controlling obesity. Controlled studies with a larger sample and follow-up longer than 16 weeks could elucidate this question.

Another relevant finding consists in CCr being significantly lower in the obese group (1.34 ± 0.83 mL/Kg/min; *p* < 0.05) compared to control (2.69 ± 1.3 mL/Kg/min). However, there was no significant difference between animals supplemented with HS tea (1.89 ± 0.89 mL/Kg/min, p > 0.05) and control. This finding suggests a nephroprotective effect of HS, in accordance with previous studies^25^^,^ 26, which demonstrate that obesity and metabolic syndrome can have a deleterious effect on kidney function.

The sodium dosage in obese animals that received HS tea (G4) was slightly higher compared to the control. Although this increase was not significant among animals that received only tea (G2), the high concentration of sodium in the composition of this tea has already been described^27^. This fact may be relevant in patients with other comorbidities associated with obesity, such as high blood pressure. However, to date, evidence to support our results is lacking in the literature.

We observed a significant higher LDL cholesterol in obese animals (G3) in comparison with the obese ones supplemented with HS tea, showing that the HS extract had a protective effect, as highlighted in other studies^28^.

In our series, the animals that received commercial rat chow and HS tea (G2) presented reduction of triglycerides levels when compared with other groups (G1, G3 and G4). Thus, demonstrating that HS tea could bring benefits in animals with and without obesity^29^. In humans, HS (*Hibiscus sabdariffa*) also presented a lipid-lowering action in individuals with metabolic syndrome, showing reduction in blood glucose, total cholesterol, triglycerides and increase in HDL, through the inhibition of pancreatic lipase and reduction of intestinal lipid absorption^29^.

When we evaluated the leptin, its levels were higher in obese groups (G3 and G4) than in controls, showing that hyperlipidic diet may have caused inflammatory process in those animals, and that HS tea could not protect them from rise of this pro-inflammatory cytokine. Despite this, there was a decrease in the absolute leptin levels in G4. According to our study outcomes, HS tea was able to decrease IL-1α levels in G2 and G4. Some authors stated that HS could reduce leptin levels by direct action on adipocytes^30^^,^ 31. The generation of pro-inflammatory factors in visceral adipose tissue can also influence alterations on the sensory function of the bladder^32^. In our study, there was no significant difference between anti-inflammatory ILs in different groups, which allows us to infer that the changes found were mainly due to the increase in pro-inflammatory adipokines.

We observed a significant decrease in thickness of detrusor muscle fibers in the obese animals supplemented with HS tea (G4). Some authors^33^ observed, in controlled trials, that animals supplemented with hyperlipidic diet presented an increase in thickness of bladder muscle fiber, and our study demonstrated the protective effect of HS tea.

In the bladder, type-I, III and IV collagens are the most common, with type I being more prevalent. Type-I fibers are more mature, and its increase is related to chronic processes that can lead to an increase in volumetric bladder load^34^. Type-III fibers represent immature collagen and are related to the disruption of the organ’s homeostasis, being synthesized in repair and fibrosis processes. According to the function of each organ, over time, they can be replaced by type-I collagen^35^.

In our study, we observed a significant decrease in the total collagen count in G4 when compared with G1 and G3. In another obesity model, using hyperlipidic diet, the authors reported increase of collagen in these animals^16^. Thus, we may imply that, in our series, HS tea could avoid fibrosis within the detrusor muscle. However, in our series, we observed a predominance of type-I and III collagens, but there was no significant difference between the groups. Therefore, it does not allow us to assess the impact of either obesity or the protective effect of HS on collagen remodeling.

Possible limitations of the study include the fact that no other morphometric analyses of the bladder were performed. Furthermore, the study of different types of collagen using more sensitive techniques and markers, such as immunohistochemistry, for example, would be more appropriate. The use of recent techniques could help us highlight differences that could not be demonstrated with the applied methodology. Likewise, an ultrastructural study of detrusor muscle cells was initially considered, in an attempt to identify changes that were not observed under optical microscopy. However, these procedures would greatly increase the costs of the research, which would preclude carrying it out with the resources currently available. Likewise, our model did not allow us to evaluate whether the morphological changes observed in the bladder were capable of inducing any changes in the functional pattern of the bladder in these animals. Furthermore, we cannot say what their behavior would be over time either, that is, whether they would remain stable or evolve into a more severe pattern over time. Finally, the use of an experimental model in rats has the advantage of allowing the researcher to control numerous variables that may interfere with the observed results, such as ensuring adequate intake of different components of the diet or obtaining different materials for analysis. However, there is no way to guarantee that the results obtained are faithfully translatable to humans.

## Conclusion

Taken together current data supports the HS tea anti-inflammatory role, with potential to reverse hyperlipidemia in rats, and to reduce deleterious effects of obesity on these animals’ bladder.

## Data Availability

All data generated or analyzed in the current study are available upon request.
